# A Stroke of Vision as One-and-a-Half Syndrome: Is It Time to Update the FAST Criteria and ABCD2 Score?

**DOI:** 10.7759/cureus.29370

**Published:** 2022-09-20

**Authors:** Mansoor Zafar, Yasmin McCafferty, Awais Sarwar, Lieze Thielemans, Bethan Davies

**Affiliations:** 1 Gastroenterology, General Internal Medicine, Royal Sussex County Hospital, University Hospitals Sussex NHS Foundation Trust, Brighton, GBR; 2 General Internal Medicine, Royal Sussex County Hospital, University Hospitals Sussex NHS Foundation Trust, Brighton, GBR; 3 Emergency Medicine, Royal Sussex County Hospital, University Hospitals Sussex NHS Foundation Trust, Brighton, GBR; 4 Internal Medicine, Royal Sussex County Hospital, University Hospitals Sussex NHS Foundation Trust, Brighton, GBR; 5 Stroke Medicine, Medicine for Elderly, Royal Sussex County Hospital, University Hospitals Sussex NHS Foundation Trust, Brighton, GBR

**Keywords:** fast, nihss (national institutes of health stroke scale), one-and-a-half syndrome, stroke, internuclear ophthalmoplegia

## Abstract

FAST (Face, Arm, Speech, Time) is the most commonly used acronym to identify a possible acute stroke. However, it fails to include visual or vestibular changes as potential hallmarks of an acute event. In this case report, we discuss a patient presenting with visual disturbances and internuclear ophthalmoplegia, with a resulting diagnosis of acute ischaemia. We discuss the associated causes, syndromes, and acute management. Though FAST is an important tool for early recognition of a possible stroke, we want to highlight the consideration of visual changes as an increasing phenomenon in an acute cerebrovascular event.

## Introduction

Internuclear ophthalmoplegia (INO) has been defined as a disorder of ocular motility, where the conjugate lateral gaze is affected due to a lesion at the medial longitudinal fasciculus (MLF) [1]. On the ipsilateral side of the lesion, the affected eye has restricted adduction. Hence, on attempting lateral gaze towards the contralateral side, the affected eye shows only minimal adduction. The contralateral eye is still able to abduct, though associated nystagmus may be seen. Convergence is generally preserved. In some cases, patients can develop convergence insufficiency resulting in diplopia, such as in our case report [1].

The most common reason for INO in older individuals is a stroke. Among the younger population, cases are linked to multiple sclerosis (MS) [1]. INO has been reported to occur in 15-52% of individuals with MS [2]. Other less common causes include Lyme disease, neurosyphilis, brain tumours, head injuries, nutritional deficiencies such as Wernicke encephalopathy, and certain drugs, such as phenothiazines, opioids, and tricyclic antidepressants [1]. Kean et al. (2005) reported that the cause of INO was 38%, MS in 34%, and rarer causes (including infection and iatrogenic tumours) in 28% of the 410 patients in a retrospective sample study spanning over 33 years [3].

## Case presentation

A 68-year-old male presented to the emergency department (ED) at a tertiary city hospital with acute onset of dizziness and vomiting that started whilst walking on the beach. He was driven to the hospital by his son and wife. His medical history included hypertension, hyperlipidaemia, triple cardiac bypass surgery 15 years ago, tinnitus, and cataract surgery in the left eye two weeks prior to his admission. There was no family history of strokes. His Rockwood (frailty) score was 2. Initial observations including blood pressure, heart rate, oxygen saturation, temperature, and Glasgow Coma Scale (GCS) score were all within normal range. An electrocardiogram (ECG) showed nil acute ischaemic changes. Chest X-ray was clear. It was noted on triage that he was FAST negative. A stroke emergency call was not put out initially. However, after reviewing by ED doctors, concerned with the patient’s ongoing neurological symptoms, the on-call stroke team was alerted 60 minutes after the patient had arrived at the department.

**Figure 1:**
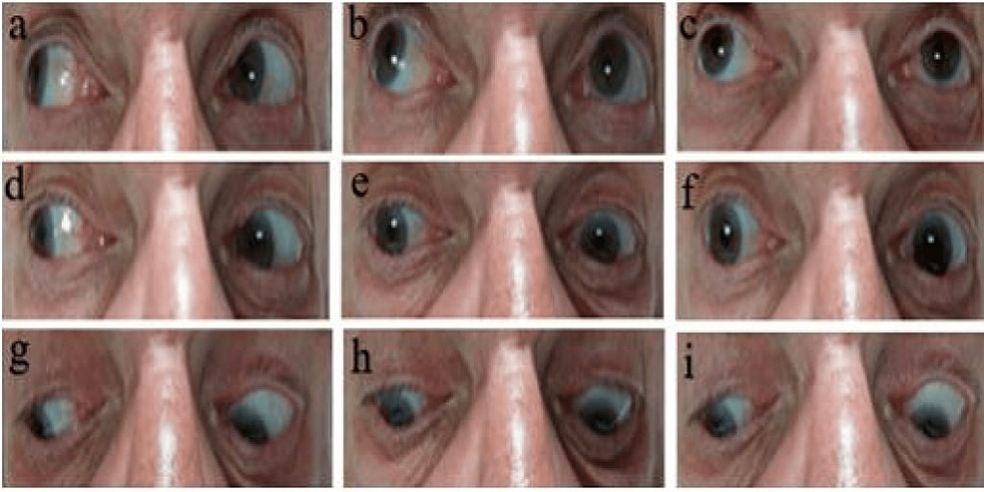
Impaired conjugate deviation in all directions. Looking up and right (a), looking up (b), looking up and left (c), looking right (d), looking straight (e), looking left (f), looking down and right (g), looking down (h), and looking down and left (i).

The patient reported double vision when looking to the right, stated seeing two images side-by-side, signifying horizontal diplopia, and on examination of cranial nerves had oculomotor changes. He was noticed to have a right eye abduction (laterally deviated) with a fixed left eye, unable to move laterally in either direction past the midline. On assessing his right lateral gaze, his right eye was abducted; however, the left eye was unable to adduct (move medially beyond the midline). Similarly, restricted movements were noticed when asked to look up and right and look down and right. When assessing the left lateral gaze, the left eye was not able to abduct, and the right eye was not able to adduct. Similarly, restricted movements were noticed on asking to look up and left and look down and left. On asking to look straight ahead, his right eye remained laterally deviated, while his left eye was unable to move nasally beyond the midline. Similar restrictions were noticed while looking up and down. Pupils were reactive to light on direct and consensual reflexes, and the accommodation reflex was minimally intact (Figure 1, Video 1).

**Video 1:** Impaired conjugate deviation in all directions; right and left and when looking up and down.

**Figure 2:**
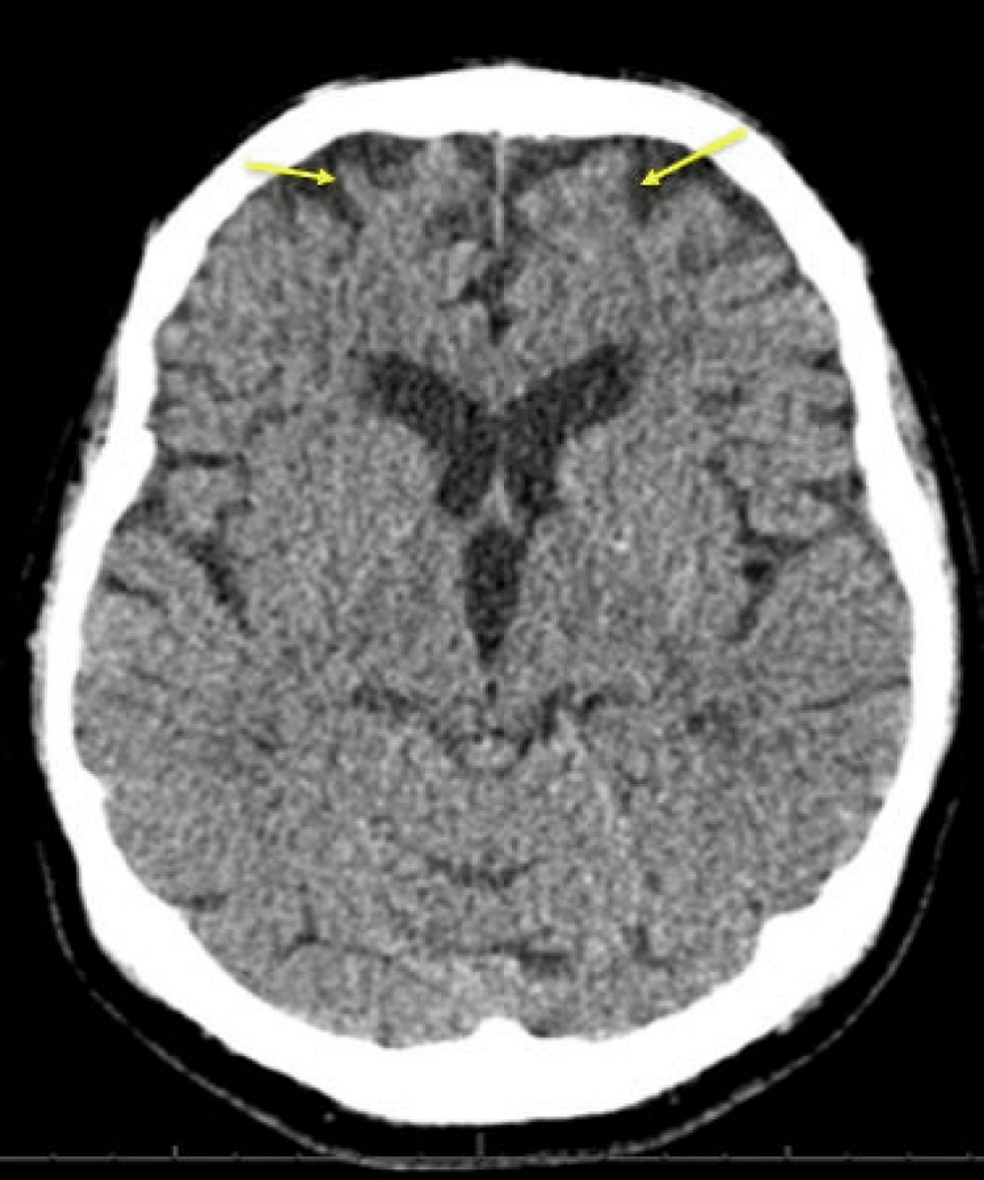
Non-contrast computed tomography of the head with nil acute findings. Normal ventricles and basal cisterns. Some atrophy of the frontal lobes appeared advanced for the patient’s age (yellow arrows).

The patient’s gait was not assessed as he declined with concerns about feeling unbalanced and symptoms of vertigo. No other sensory, motor, cerebellar, or extrapyramidal impairment was noticed on a neurological examination of the limbs. His National Institutes of Health Stroke Scale (NIHSS) score was 2. He underwent an urgent non-contrast computed tomography (CT) of the head that did not show any bleeding, space-occupying lesions, or any acute ischaemic changes (Figure 2).

An impression of ‘one-and-a-half syndrome’ was made with a query of involvement of the left MLF and left para-pontine reticular formation (PPRF). The case was discussed with the on-call stroke consultant, and he was treated with high-dose antiplatelet therapy and oral aspirin (300 mg) once daily for 14 days along with 30 mg oral lansoprazole (a proton-pump inhibitor). He was admitted to the stroke ward for close monitoring. He underwent blood tests and the sample was sent to assess for stroke risk factors, which showed mild derangement in lipid profile and slightly elevated haemoglobin A1c (Hb1Ac), though otherwise unremarkable. C-reactive protein was 2 mg/dL, haemoglobin 135 g/L, mean corpuscular volume 88.8 fL, platelet count 221 ×10^9^/L, Na 140, K 4.4, creatinine 95 µmol/L, estimated glomerular filtration <60, and Hb1Ac 44 (<42) (Table 1).

**Table 1:** Serum lipid profile. Source: Laboratory, Royal Sussex County Hospital, Brighton, UK. HDL: high-density lipoprotein

Lipid profile, serum	Units	Range	Serum levels of patient
Serum cholesterol	mmol/L	0–5	5.3
Serum HDL cholesterol	mmol/L	1–3	1.0
Serum cholesterol: HDL ratio	-	-	5.3
Serum triglyceride	mmol/L	0–2	3.5
Serum non-HDL cholesterol	mmol/L	0–3.9	4.3

**Figure 3:**
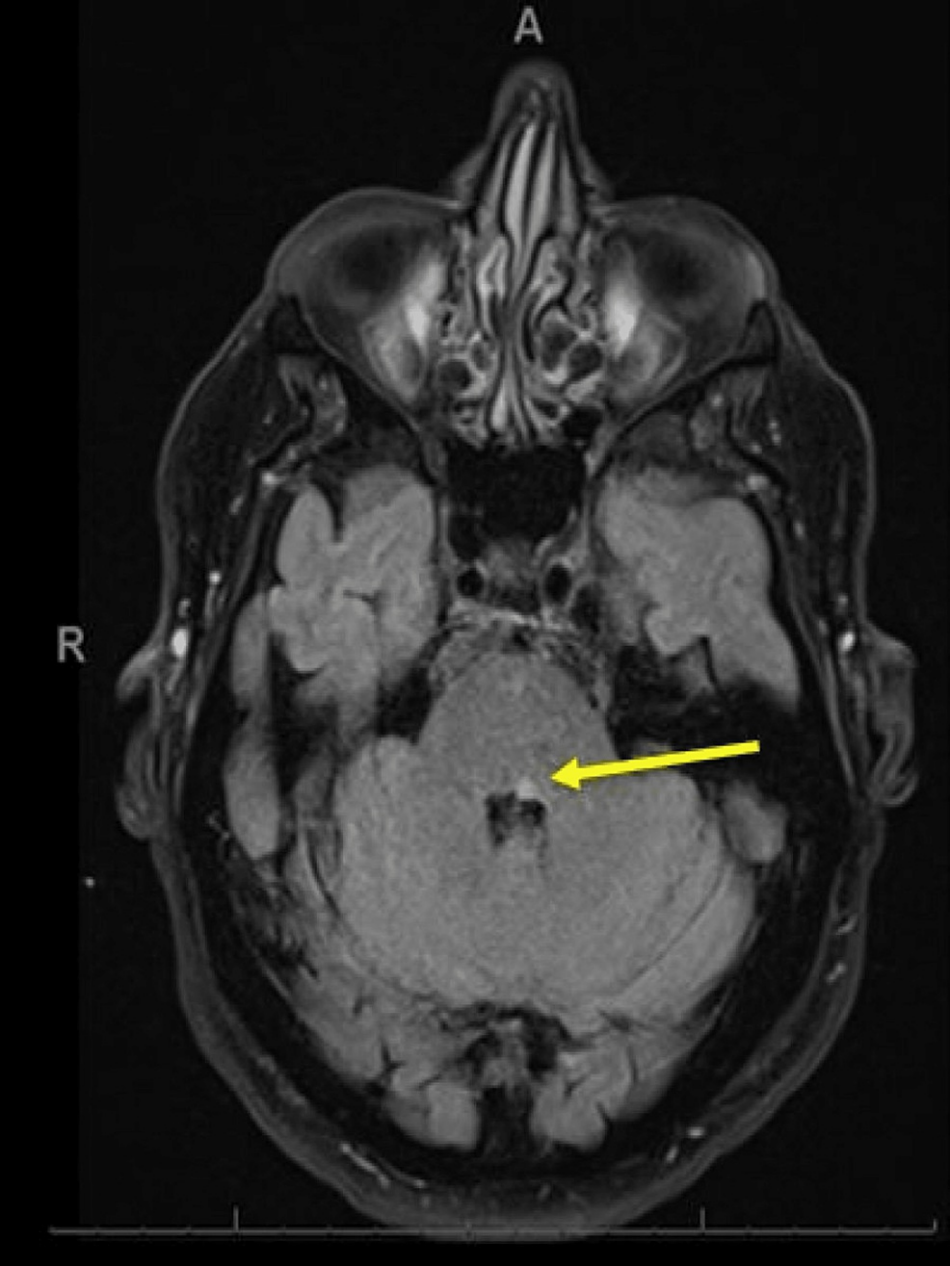
Magnetic resonance imaging of the brain (axial view): a tiny focus of restricted diffusion in the region of the left medial longitudinal fasciculus which in this age group and context likely represents a small acute infarct (yellow arrow).

**Figure 4:**
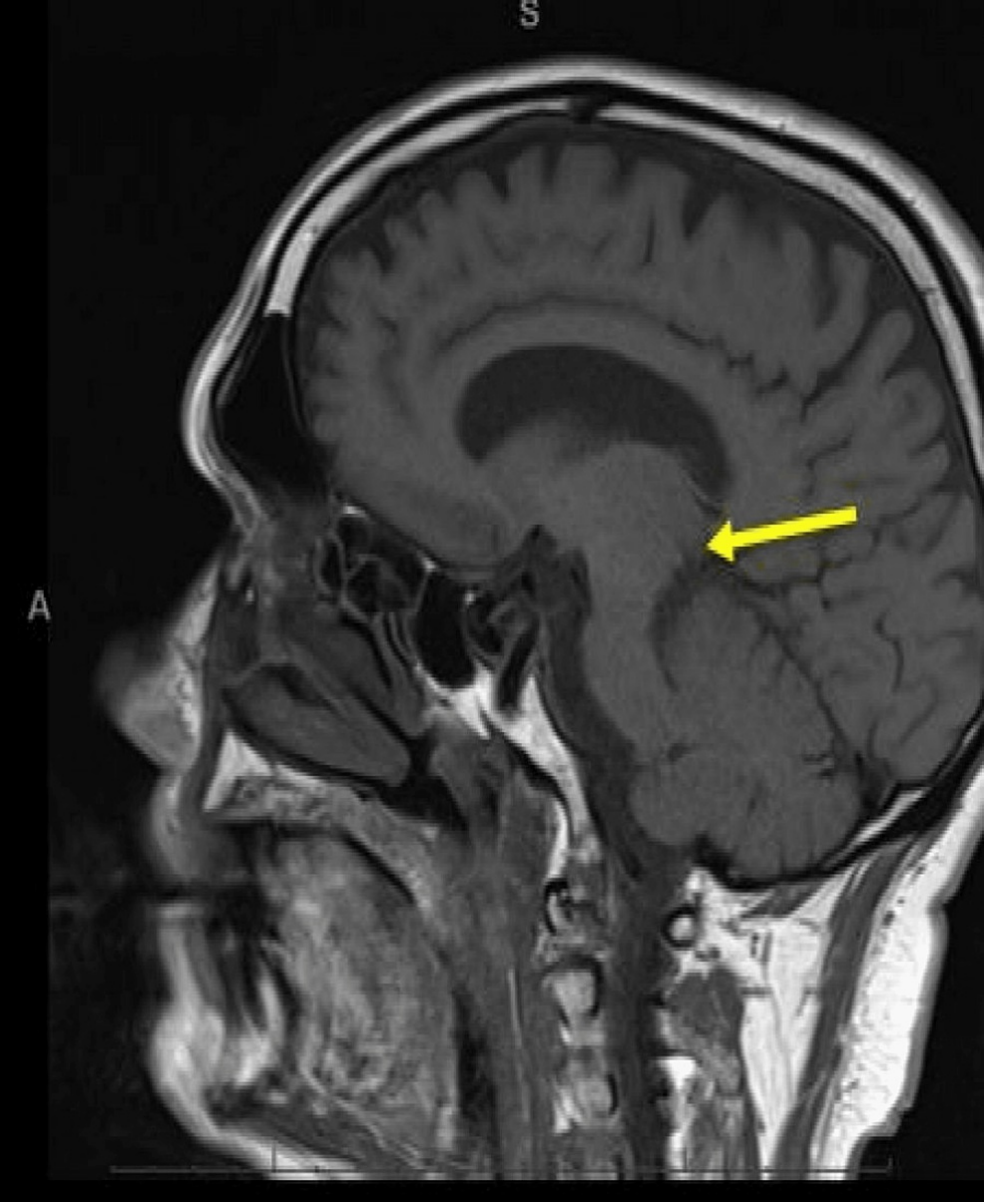
Magnetic resonance imaging of the brain (sagittal view): re-demonstration of restricted diffusion in the left paramedian tegmentum on diffusion-weighted imaging (yellow arrow).

**Figure 5:**
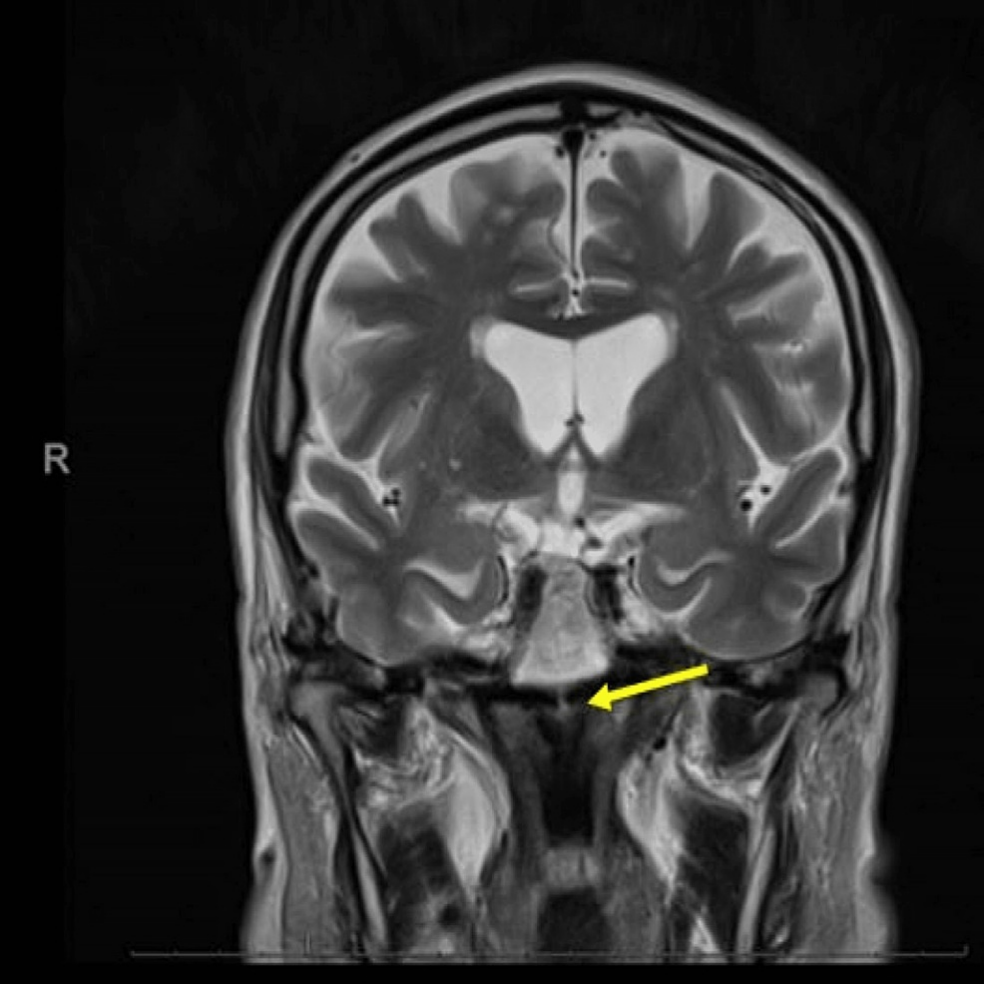
Magnetic resonance imaging of the brain (coronal view): re-demonstration of restricted diffusion in the left paramedian tegmentum (yellow arrow).

The following day, the patient underwent magnetic resonance imaging (MRI) of the brain to investigate infarct burden. This confirmed an acute ischaemic stroke in the MLF (Figures 3-5).

Most of the patient’s symptoms started to resolve after 24 hours of admission and he was discharged home with instructions to start a second anti-platelet (clopidogrel 75 mg once a day indefinitely) after completing 14 days of high-dose aspirin, along with a proton-pump inhibitor and a statin. Safety-net advice was to contact emergency services 999 in the United Kingdom (911 in the United States) immediately if experiencing any new FAST symptoms, including vision difficulties. He was booked for outpatient stroke clinic follow-up for further investigations, including a 24-hour ECG, ultrasound carotid Doppler (to assess if there was a degree of carotid artery stenosis), and echocardiogram (to rule out any structural cardiac abnormalities). The patient was advised to inform the vehicle licensing authority (DVLA) in the United Kingdom and to refrain from driving till reviewed by his general practitioner (family doctor) in the community.

## Discussion

Although INO has classically been described as a disorder of lateral conjugate gaze, it has been known to present as five variants or different syndrome complexes [4].

Wall-eyed bilateral internuclear ophthalmoplegia (WEBINO) which is associated with bilateral damage to the MLF [5]. Both eyes are seen to be looking at opposite walls (both eyes turned away from the nose), hence, the term exotropia [6]. Wall-eyed monocular internuclear ophthalmoplegia (WEMINO), which is similar to WEBINO but unilateral, with one eye looking away from the nose (exotropia), and is less common [7]. The one-and-a-half syndromeinvolves the MLF and PPRF in the dorsal tegmentum of the caudal pons, with or without the involvement of cranial nerve VI on the ipsilateral side [8]. The eye on the contralateral side is abducted with abduction nystagmus and has an impaired ability to adduct [9]. The half-and-half syndrome involves the MLF and cranial nerve VI without the involvement of the VI nerve nucleus [9]. Finally, in reverse INO or Lutz posterior INO, instead of adduction, an abduction deficit occurs with varying localisation to the midbrain [9], pontine MLF [9], rostral pons [10], or PPRF [10].

The NIHSS is a 15-item neurological examination stroke scale used to assess the effect of acute cerebral stroke. The score outcome is stratified as very severe (>25), severe (15-24), mild-to-moderately severe (5-14), and mild (1-4) [11]. With 42 being the highest score possible, the higher the NIHSS score, the more impaired a stroke patient is likely to be [12].

An NIHSS score of 4 or less is commonly used as an exclusion criterion for thrombolysis treatment based on the original NINDS tPA trial exclusion of minor, non-disabling symptoms [13,14]. On the contrary, intravenous thrombolysis is contraindicated usually with an NIHSS score >25 [15]. Our patient was not a candidate for thrombolysis with an NIHSS score of 2 and was managed medically.

The acronym FAST (Facial drooping, Arm weakness, Speech difficulties, and Time) was first introduced in the United Kingdom in 1998 [16]. However, various publications have recommended changes to it, including FASTER [16], FAST-V or V-Fast [17], or BE-FAST [18]. It is important to acknowledge the inclusion of vision and associated balance as an increasingly occurring phenomenon in stroke, and therefore, the acronym FAST should be updated to reflect this. According to Rowe et al., the incidence of visual problems during an acute stroke is high, affecting more than half of survivors. [19]. We recommend a new mnemonic for acute stroke as ‘Be Very FAST’ (‘Be’, balance; ‘Very’, vision; ‘F’, facial drooping; ‘A’, arm weakness; ‘S’, speech difficulties; and ‘T’, time elapsed window following an event) (Table 2).

**Table 2:** Infographic recommendation for the new ‘Be Very FAST’ criteria for acute stroke.

Abbreviation	Mnemonic: ‘Be Very FAST’	Description
Be	Balance or coordination	Sudden impairment in balance or impaired coordination
Very	Vision	Sudden loss of vision or complaint of blurry or double vision
F	Face	Sudden facial drooping
A	Arm	Weakness or drift of the arm
S	Speech	Sudden impairment in speech including slurring, impairment in speech or repetition of a phrase or phrases
T	Time	Act to call 911 (USA) or 999 (UK) or transport to hospital emergency department

We also recommend a new scoring system of ‘ABCD2E’ while formulating the risk of stroke in patients presenting with a transient ischaemic attack instead of the older scoring system of ABCD2 (Table 3) again highlighting the need for the inclusion of visual symptoms in patients presenting with symptoms of a transient ischaemic attack, as proposed by Zafar et al. [20,21].

**Table 3:** Infographic recommendation for the new ABCD2E score. The current ABCD2 score with score ranging from 0 to 7. Recommended ABCD2E with score ranging from 0 to 8. Adapted from the current ABCD2 score [21]. SBP: systolic blood pressure; DBP: diastolic blood pressure

Abbreviation (Mnemonic: ABCD2E)	Parameters	Score
A	Age (years)	
	> 60	1
B	BP (mmHg)	
	SBP > 140 or DBP > 90	1
C	Clinical features	
	Unilateral weakness	2
	Speech disturbance without weakness	1
D	Duration of symptoms (minutes)	
	≥ 60	2
	10–59	1
D^2^	Diabetes	1
E	Eye signs with sudden transient vision impairment including blurry vision, double vision or loss of vision	1

It will be interesting to see more case reports concerning visual findings in patients with acute stroke or transient ischaemic attacks. Perhaps it is time to update the FAST criteria to a newer Be Very FAST criteria for acute stroke and ABCD2 to a newer ABCD2E scoring system for patients with transient ischaemic attacks.

## Conclusions

INO is a disorder of conjugate lateral gaze caused by a lesion at the MLF. INO is most commonly caused by stroke in older patients and by MS in younger patients. There are five variants of INO, and this case report illustrates the presentation of one variant called the one-and-a-half syndrome. This case report highlights the significance of recognising visual symptoms as a hallmark of stroke. Public health campaigns and slogans such as FAST do not include visual symptoms, which could delay the layperson from recognising these symptoms as an important indicator of stroke, preventing timely presentation to the ED.

Introducing a new or altered mnemonic to guide the public in recognising strokes with varied presentations may lead to better health outcomes in the future. We recommend a new mnemonic for acute stroke as ‘Be Very FAST’ (‘Be’, balance; ‘Very’, vision; ‘F’, facial drooping; ‘A’, arm weakness; ‘S’, speech difficulties; and ‘T’, time elapsed window following the event).

Lastly, future studies could look at updating the ABCD2 scoring utilised to predict future stroke in patients with transient ischaemic attacks with the inclusion of vision. We recommend a new scoring system of ‘ABCD2E’ while formulating the risk of stroke in patients presenting with the transient ischaemic attack instead of the older scoring system of ABCD2.
